# Mortality risk in patients with autosomal dominant polycystic kidney disease

**DOI:** 10.1186/s12882-024-03484-3

**Published:** 2024-02-16

**Authors:** Deirdre Mladsi, Xiaolei Zhou, Gregory Mader, Myrlene Sanon, Jinyi Wang, Christine Barnett, Cynthia Willey, Stephen Seliger

**Affiliations:** 1 RTI Health Solutions, Research Triangle Park, NC USA; 2 Otsuka Pharmaceutical Development & Commercialization, Inc., Princeton, NJ USA; 3 University of Rhode Island, Kingston, RI USA; 4 University of Maryland School of Medicine, Baltimore, MD USA

**Keywords:** Autosomal dominant polycystic kidney disease, End-stage renal disease, Mortality

## Abstract

**Background:**

Autosomal dominant polycystic kidney disease (ADPKD) is the leading inheritable cause of end-stage renal disease (ESRD). Mortality data specific to patients with ADPKD is currently lacking; thus, the aim of this study was to estimate mortality in patients with ADPKD.

**Methods:**

We analyzed data from the United States Renal Data System (USRDS) for patients with ADPKD available during the study period of 01/01/2014–12/31/2016, which included a cohort of patients with non-ESRD chronic kidney disease (CKD) and a cohort of patients with ESRD. Mortality rates with 95% confidence intervals (CIs) were calculated overall and by age group, sex, and race for the full dataset and for a subset of patients aged ≥ 65 years. Adjusted mortality hazard ratios (HRs) were calculated using Cox regression modeling by age group, sex, race, and CKD stage (i.e., non-ESRD CKD stages 1–5) or ESRD treatment (i.e., dialysis and transplant).

**Results:**

A total of 1,936 patients with ADPKD and non-ESRD CKD and 37,461 patients with ADPKD and ESRD were included in the analysis. Age-adjusted mortality was 18.4 deaths per 1,000 patient-years in the non-ESRD CKD cohort and 37.4 deaths per 1,000 patient-years in the ESRD cohort. As expected, among the non-ESRD CKD cohort, patients in CKD stages 4 and 5 had a higher risk of death than patients in stage 3 (HR = 1.59 for stage 4 and HR = 2.71 for stage 5). Among the ESRD cohort, patients receiving dialysis were more likely to experience death than patients who received transplant (HR = 2.36). Age-adjusted mortality among patients aged ≥ 65 years in the non-ESRD CKD cohort was highest for Black patients (82.7 deaths per 1,000 patient-years), whereas age-adjusted mortality among patients aged ≥ 65 years in the ESRD cohort was highest for White patients (136.1 deaths per 1,000 patient-years).

**Conclusions:**

Mortality rates specific to patients aged ≥ 65 years suggest racial differences in mortality among these patients in both non-ESRD CKD and ESRD cohorts. These data fill an important knowledge gap in mortality estimates for patients with ADPKD in the United States.

**Supplementary Information:**

The online version contains supplementary material available at 10.1186/s12882-024-03484-3.

## Background

Autosomal dominant polycystic kidney disease (ADPKD) is a dominantly inherited systemic disease characterized by uncontrolled development and growth of multiple renal cysts that lead to gradual enlargement of the kidneys [1]. Over time, ADPKD results in a progressive loss of renal function and, ultimately, end stage renal disease. ADPKD is the leading inheritable cause of ESRD and the 4 ^th^ leading cause of ESRD overall [2–4]. Common complications and comorbidities of ESRD include a mortality rate of 20% to 50% (2-year rate), coronary heart disease, peripheral vascular disease, and hypertension [5]. Both maintenance dialysis and complications associated with ESRD can significantly impact the quality of life for patients [6–10] while also posing a substantial burden to healthcare systems [11–13].

The United States Renal Data System (USRDS), funded by the National Institute of Diabetes and Digestive and Kidney Diseases, is a national data system that collects, analyzes, and distributes information about chronic kidney disease (CKD) and ESRD in the United States (US) [14]. While the USRDS has reported mortality for patients with non-ESRD CKD [15], mortality data specific to patients with ADPKD is currently not available. An unadjusted mortality rate of ~ 16 per 100 patient-years for patients aged ≥ 65 years with ESRD and ADPKD has been estimated with the use of USRDS data from 2001 through 2010 [16]. However, given advancements in treatment and guidelines over the last decade and the availability of novel treatments to slow ADPKD progression, more recent mortality estimates are needed in both ESRD and non-ESRD CKD. Moreover, although research has suggested racial differences among patients with CKD in access to treatment, incidence, progression, and mortality [4, 16–19], findings specific to patients with ADPKD are lacking.

A current understanding of mortality and racial differences associated with mortality is needed for patients with ADPKD with non-ESRD CKD and those who have progressed to ESRD. Our study sought to estimate mortality in ADPKD in non-ESRD CKD (i.e., patients who had not received treatment of dialysis or transplant) and in ESRD (i.e., patients who received treatment of dialysis or transplant) overall and by sex, race, and age group for the overall population and a subset of patients aged ≥ 65 years. We also compared risk of mortality among patients with non-ESRD CKD by CKD stages 1 through 5, among patients with ESRD by treatment of dialysis or transplant, and among patients in both cohorts by age group, sex, and race.

## Methods

### Study design and data sources

This retrospective observational study analyzed data from the USRDS for patients with ADPKD available during the study period of 1 January 2014 to 31 December 2016. This analysis used deidentified data acquired from a national data system and accordingly received a “not human research” determination from the RTI Institutional Review Board (Federalwide Assurance Number 3331).

The USRDS uses Medicare claims data to identify patients with CKD according to the International Classification of Diseases, Ninth Revision, Clinical Modification (ICD-9-CM) and the International Classification of Diseases, Tenth Revision, Clinical Modification (ICD-10-CM) codes and makes datasets available to researchers for yearly cohorts of patients diagnosed with CKD within the Medicare 5% database (a random sample of 5% of the entire Medicare population) [20, 21]. With respect to CKD, the USRDS contains data from the Medicare 5% sample for patients with CKD aged ≥ 65 years and patients with CKD, regardless of age, who otherwise qualify for Medicare. Therefore, due to the restrictions of Medicare, the data for earlier-stage CKD (prior to ESRD) were limited to patients aged ≥ 65 years unless patients otherwise qualified for Medicare while having CKD.

In addition to Medicare data, the USRDS database contains data collected from the ESRD Medical Evidence Report form [20] for the entire US ESRD population and the ESRD Death Notification form [21]. The latter is required to be submitted by dialysis or transplant providers when patients with ESRD die and is the primary source of death information for such patients. The USRDS also uses other supplemental data sources, such as the United Network for Organ Sharing (UNOS) transplant data, census data, and data from the ESRD networks.

### Patient cohorts and follow-up

Criteria for inclusion in the ADPKD non-ESRD CKD study cohort and the ADPKD ESRD study cohort are presented in Table 1. For the non-ESRD CKD study cohort, the follow-up (i.e., patient-time at risk) started at the later date of the start of the study period (1 January 2014) or the date of the first ADPKD diagnosis code. It ended on the earliest date of the following: death, end of follow-up or end of study period (31 December 2016), or first use of ESRD services. For patients in the ESRD study cohort, the follow-up started at the later date of start of the study period (1 January 2014) or first use of ESRD services. It ended on the earlier date of the following: end of study period (31 December 2016) or death.

**Table 1:** Eligibility criteria

Cohort	Inclusion criteria
ADPKD Non-ESRD CKD	▪ Had at least 2 records with an ADPKD diagnosis code (ICD-9-CM codes: 753.13, 753.12; ICD-10-CM codes: Q61.2, Q61.3) in the CKD database across all available years
	▪ Was included in the 2014, 2015, or 2016 CKD cohort, as defined by the researcher’s guide [22] as having met both of the following criteria: • For the entire year, the patient was Part A and Part B entitled and was not enrolled in a health maintenance organization • The patient had at least 1 CKD diagnosis code from an inpatient or home health or skilled nursing facility SAF, or at least 2 CKD diagnosis codes from a physician or supplier, durable medical equipment, or outpatient SAF with different claim dates
	▪ The patient was alive and did not receive ESRD services as of 1 January 2014
ADPKD ESRD	▪ Had at least 1 ADPKD diagnosis code (ICD-9-CM codes: 753.13, 753.12; ICD-10-CM codes: Q61.2, Q61.3) reported in the ESRD Medical Evidence Report form [20] as the primary cause of renal failure
	▪ First ESRD service date was on or before 1 December 2016 so that each patient would have at least 1 month of follow-up before the end of the study period
	▪ Was alive (based on the death date) as of 1 January 2014
	▪ Had no flag for a data issue

*ADPKD* Autosomal dominant polycystic kidney disease, *CKD* Chronic kidney disease, *ESRD* End-stage renal disease, *ICD-9-CM* International Classification of Diseases, Ninth Edition, Clinical Modification, *ICD-10-CM* International Classification of Diseases, Tenth Edition, Clinical Modification, *SAF* Standard Analytic File (CMS)

The CKD stages and ESRD treatments (i.e., dialysis, transplant) were defined at study entry (i.e., the start of patient-time at risk defined above). The CKD stages were defined based on ICD-9-CM and ICD-10-CM codes in claims. Patients who received a transplant on or before the study entry date were included in the transplant group. Patients who received dialysis on or before the study entry date were included in the dialysis group. Patients who received both transplant and dialysis on or before the study entry date were included in the transplant group.

### Analyses

Demographic characteristics were summarized by using descriptive statistics. Mortality rate was calculated as the number of deaths occurring during the patient-time at risk divided by the total patient-time at risk. Mortality rates along with 95% confidence intervals (CIs) were calculated overall, by age group at study entry, by sex, and by race in the overall population. The exact CIs were calculated by using the relationship between the Poisson distribution and Chi-square distribution as described in Dobson et al. [23]. Age-adjusted mortality was calculated based on the US general population [24]. Adjusted mortality hazard ratios (HRs) were calculated using Cox models that included age group, sex, race, and CKD stage (for patients with non-ESRD CKD) or ESRD treatment (i.e., dialysis and transplant for patients with ESRD). When a category had zero events, Firth penalized maximum likelihood estimation was used to reduce bias in the estimates [25]. All adjusted HRs were reported as point estimates with 95% CIs.

Similar analyses were conducted for the subset of patients aged ≥ 65 years. This subset included patients in the ADPKD non-ESRD CKD or ESRD cohort who reached 65 years before the end of the study period and excluded patients without eligible follow-up. Mortality rates along with 95% CIs were calculated overall, by age group, by sex, by race, and by the combination of sex and race. For a small proportion of the patients, the follow-up spanned 2 age groups during the study period; for these patients, their follow-up time was partitioned and allocated across the 2 age groups; therefore, the mortality analyses for the subset of patients aged 65 and older is based on the number of records rather than the number of patients. Age-adjusted mortality rates were standardized to the US population age distributions for patients aged ≥ 65 years [26]. Adjusted HRs were estimated for the subset of patients aged ≥ 65 years at study entry with non-ESRD CKD and ESRD.

## Results

### Demographic characteristics

**Fig. 1:**
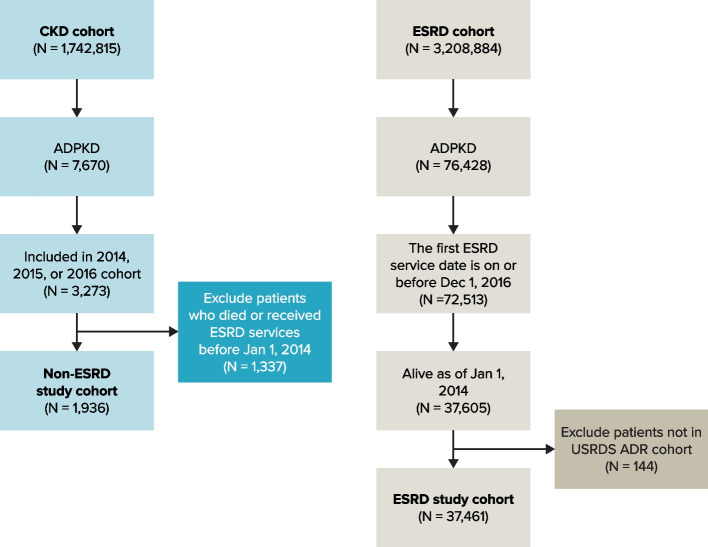
Selection of patients for the ADPKD Non-ESRD CKD and ESRD Cohorts ADR = Annual Data Report; ADPKD = autosomal dominant polycystic kidney disease; CKD = chronic kidney disease; ESRD = end-stage renal disease; USRDS = United States Renal Data System. Note: USRDS provided a flag variable in the ESRD database to indicate a data issue and stated that they did not include these records in the ADR; therefore, the current analysis also excluded these records. As shown in the figure, this resulted in 144 patients being excluded from the ESRD study cohort

Of 1,742,815 patients with CKD in the USRDS database, 1,936 patients (0.1%) met the criteria for inclusion in the study cohort of ADPKD patients with non-ESRD CKD (Fig. 1). Of 3,208,884 patients with ESRD in the USRDS database, 37,461 were patients with a diagnosis of ADPKD whose records fulfilled study criteria for inclusion (1.2%). Among patients with non-ESRD CKD, 79.6% were 65 years and older at study initiation (Table 2). Among patients with ESRD, 80.4% were from 45 to 74 years of age at study initiation, and those aged 55 to 64 years comprised 34.0% of the study cohort (Table 2). For both cohorts, approximately 53% were male and the majority were White (79.6% of the non-ESRD CKD cohort; 73.8% of the ESRD cohort).

**Table 2:** Demographic characteristics of patients included in the ADPKD study cohorts

Characteristics	Non-ESRD CKD study cohort (N = 1,936)	ESRD study cohort (N = 37,461)
Age		
Mean (SD)	71.4 (13.2)	59.2 (11.8)
Median	72.0	59.0
Min, max	2, 107	0, 99
Age group, n (%)		
< 18 years	2 (0.1%)	38 (0.1%)
18–24 years	3 (0.2%)	133 (0.4%)
25–34 years	26 (1.3%)	683 (1.8%)
35–44 years	48 (2.5%)	2,915 (7.8%)
45–54 years	138 (7.1%)	8,756 (23.4%)
55–64 years	178 (9.2%)	12,728 (34.0%)
65–74 years	688 (35.5%)	8,635 (23.1%)
75–84 years	564 (29.1%)	3,060 (8.2%)
85 years and older	289 (14.9%)	513 (1.4%)
Sex, n (%)		
Male	1,030 (53.2%)	19,999 (53.4%)
Female	906 (46.8%)	17,462 (46.6%)
Race, n (%)		
White	1,542 (79.6%)	27,637 (73.8%)
Black	271 (14.0%)	4,504 (12.0%)
Hispanic	36 (1.9%)	3,924 (10.5%)
Asian	36 (1.9%)	1,018 (2.7%)
Other or unknown^ a^	51 (2.6%)	378 (1.0%)

*ADPKD* Autosomal dominant polycystic kidney disease, *CKD* Chronic kidney disease, *ESRD* End-stage renal disease, *SD* Standard deviation, *USRDS* United States Renal Data System

^a^In addition to “White,” “Black,” “Hispanic,” and “Asian” in the non-ESRD CKD USRDS dataset, “race” also included “Native American,” “Other,” and “Unknown,” which have been grouped in this table as “Other or unknown.” In addition to “White,” “Black/African American” (referred to as “Black” in this table), “Hispanic,” and “Asian” in the ESRD USRDS dataset, “race” also included “American Indian or Alaska Native,” “Native Hawaiian or Pacific Islander,” “Other or Multiracial,” and Unknown, which have been grouped in this table as “Other or unknown.”

In the subset of patients aged ≥ 65 years, there were 1,696 records with a total of 3,059.1 person-years in the non-ESRD CKD analysis set, and 16,583 records with a total of 32,041.8 person-years in the ESRD analysis set (Table 3). In this subset of patients, there was a lower percentage of records for patients in the 65- to 74-year age range in the non-ESRD CKD analysis set compared with the ESRD analysis set (41.3% vs. 67.4%). Conversely, there was a modestly higher percentage of male patient-records in the non-ESRD analysis set compared with the ESRD analysis set (54.3% vs. 50.1%). White patients comprised the majority of both analysis sets (81.5% of non-ESRD CKD patient-records and 77.3% of ESRD patient-records).

**Table 3:** Demographic characteristics in the ADPKD analysis set for age ≥ 65 years

Characteristics	Records in Non-ESRD CKD analysis set (N = 1,696)	Records in ESRD analysis set (N = 16,583)
Total person-years	3,059.1	32,041.8
Age group, n (%)		
65–74 years	701 (41.3%)	11,183 (67.4%)
75–84 years	646 (38.1%)	4,523 (27.3%)
≥ 85 years	349 (20.6%)	877 (5.3%)
Sex, n (%)		
Male	921 (54.3%)	8,307 (50.1%)
Female	775 (45.7%)	8,276 (49.9%)
Race, n (%)		
White	1,382 (81.5%)	12,824 (77.3%)
Black	213 (12.6%)	1,809 (10.9%)
Hispanic	24 (1.4%)	1,339 (8.1%)
Asian	35 (2.1%)	470 (2.8%)
Other or unknown^ a^	42 (2.5%)	141 (0.9%)

*ADPKD* Autosomal dominant polycystic kidney disease, *CKD* Chronic kidney disease, *ESRD* End-stage renal disease, *SD* Standard deviation, *USRDS* United States Renal Data System.32,

^a^In addition to “White,” “Black,” “Hispanic,” and “Asian” in the non-ESRD CKD USRDS dataset, “race” also included “Native American,” “Other,” and “Unknown,” which have been grouped in this table as “Other or unknown.” In addition to “White,” “Black/African American” (referred to as “Black” in this table), “Hispanic,” and “Asian” in the ESRD USRDS dataset, “race” also included “American Indian or Alaska Native,” “Native Hawaiian or Pacific Islander,” “Other or Multiracial,” and Unknown, which have been grouped in this table as “Other or unknown.”

### Mortality

**Fig. 2:**
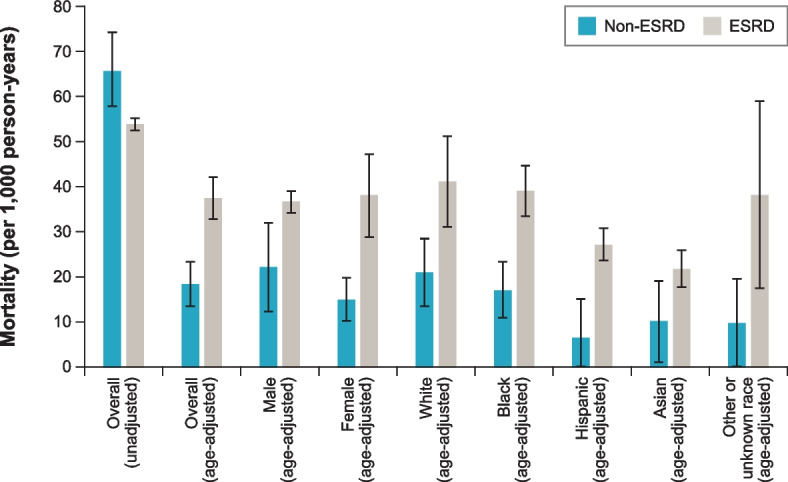
Mortality among patients with ADPKD overall, by sex, and by race ADPKD = autosomal dominant polycystic kidney disease; ESRD = end-stage renal disease. Note: In addition to “White,” “Black,” “Hispanic,” and “Asian” in the non-ESRD CKD USRDS dataset, “race” also included “Native American,” “Other,” and “Unknown,” which have been grouped in this figure as “Other or unknown.” In addition to “White,” “Black/African American” (referred to as “Black” in this figure), “Hispanic,” and “Asian” in the ESRD USRDS dataset, “race” also included “American Indian or Alaska Native,” “Native Hawaiian or Pacific Islander,” “Other or Multiracial,” and “Unknown,” which have been grouped in this figure as “Other or unknown.”

Mortality for the non-ESRD CKD cohort (unadjusted 65.6 deaths per 1,000 patient-years) was higher than that for the ESRD cohort (unadjusted 53.9 per 1,000 patient-years) (Fig. 2). Conversely, age-adjusted mortality for the non-ESRD CKD cohort (18.4 deaths per 1,000 patient-years) was lower than mortality for the ESRD cohort (37.4 deaths per 1,000 patient-years). Within the non-ESRD CKD cohort, mortality was highest for patients aged ≥ 85 years (159.0 deaths per 1,000 patient-years) and was higher for males compared with females (unadjusted 76.9 deaths vs. 53.1 deaths per 1,000 patient-years) (Table S1, Supplementary Material, Additional file 1). After the results were adjusted for age, mortality remained higher for males than for females (22.2 deaths vs. 15.0 deaths per 1,000 patient-years), but the difference was attenuated. Within the ESRD cohort, mortality was highest for patients aged ≥ 85 years (327.5 deaths per 1,000 patient-years) and was similar for males (unadjusted 54.7 deaths and age-adjusted 36.6 deaths per 1,000 patient-years) and females (unadjusted 53.0 deaths and age-adjusted 38.1 deaths per 1,000 patient-years) (Table S1, Supplementary Material, Additional file 1).

**Fig. 3:**
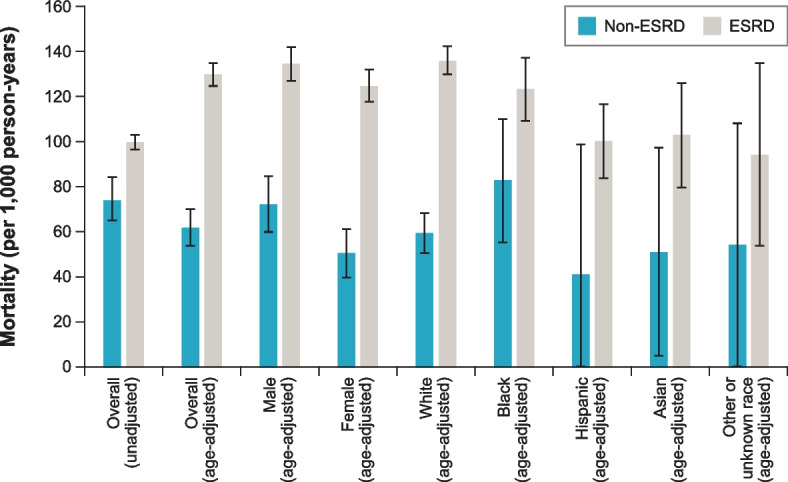
Mortality among patients with ADPKD aged ≥ 65 years overall, by sex, and by race ADPKD = autosomal dominant polycystic kidney disease; ESRD = end-stage renal disease. Note: In addition to “White,” “Black,” “Hispanic,” and “Asian” in the non-ESRD CKD USRDS dataset, “race” also included “Native American,” “Other,” and “Unknown,” which have been grouped in this figure as “Other or unknown.” In addition to “White,” “Black/African American” (referred to as “Black” in this figure), “Hispanic,” and “Asian” in the ESRD USRDS dataset, “race” also included “American Indian or Alaska Native,” “Native Hawaiian or Pacific Islander,” “Other or Multiracial,” and “Unknown,” which have been grouped in this figure as “Other or unknown.”

Among the subset of patients aged ≥ 65 years, age-adjusted mortality in the non-ESRD CKD cohort was highest for Black patients (82.7 deaths per 1,000 patient-years); however, age-adjusted mortality in the ESRD cohort was highest for White patients (136.1 deaths per 1,000 patient-years) (Fig. 3). When compared with White, Black, and Asian patients, Hispanic patients had the lowest age-adjusted mortality in both the non-ESRD CKD (unadjusted 57.2 deaths and age-adjusted 41.4 deaths per 1,000 patient-years) and ESRD (unadjusted 80.0 deaths and age-adjusted 100.3 deaths per 1,000 patient-years) cohorts (Table S2, Supplementary Material, Additional file 1). Overall unadjusted and age-adjusted mortality was lower in the non-ESRD CKD cohort (unadjusted 74.2 deaths and age-adjusted 61.9 deaths per 1,000 patient-years) compared with the ESRD cohort (unadjusted 99.8 deaths and age-adjusted 129.6 deaths per 1,000 patient-years) (Table S2, Supplementary Material, Additional file 1).

Among the subset of patients aged ≥ 65 years and within the non-ESRD CKD cohort, deaths were higher for males (unadjusted 85.7 deaths and age-adjusted 72.2 deaths per 1,000 patient-years) than for females (unadjusted 60.8 deaths and age-adjusted 50.4 deaths per 1,000 patient-years) and, among age groups, deaths were highest in the oldest age group of ≥ 85 years (unadjusted 159.7 deaths per 1,000 patient-years) (Table S2, Supplementary Material, Additional file 1). Similarly, in the ESRD cohort, deaths were higher for males (unadjusted 104.4 deaths and age-adjusted 134.4 deaths per 1,000 patient-years) than for females (unadjusted 95.3 deaths and age-adjusted 124.8 deaths per 1,000 patient-years) and, among age groups, were highest in the oldest age group of ≥ 85 years (unadjusted 340.6 deaths per 1,000 patient-years).

### Mortality risk

Within the non-ESRD CKD cohort, there was a graded increase in risk of death across higher stages of CKD (Table 4). Patients aged 75 to 84 years (HR = 1.70, *P* = 0.0112) and ≥ 85 years (HR = 3.45, *P* < 0.0001) had a statistically significantly higher risk of death than patients aged 65 to 74 years (reference group). Finally, there were no statistically significant differences in mortality risk between racial groups within this cohort (*P* ≥ 0.05 for all comparisons). Within the ESRD cohort, patients receiving dialysis were more likely to experience death than patients who received transplant (HR = 2.36, *P* < 0.0001; Table 4). Patients aged 75 to 84 years (HR = 1.75, *P* =  < 0.0001) and ≥ 85 years (HR = 2.77, *P* < 0.0001) had a statistically significantly higher risk of death than patients aged 65 to 74 years (reference group). Patients in the younger age groups had a statistically significantly lower risk of death (*P* < 0.001 for all groups) than patients aged 65 to 74 years (reference group), except for patients aged < 18 years (HR = 0.15, *P* = 0.0562). Female patients were statistically significantly less likely to experience death than male patients (HR = 0.91, *P* = 0.0006). Black (HR = 0.92, *P* = 0.0492), Hispanic (HR = 0.71, *P* < 0.0001), and Asian (HR = 0.63, *P* < 0.0001) patients had a statistically significantly lower risk of death than White patients.

**Table 4:** Adjusted hazard ratios among ADPKD patients overall

Non-ESRD CKD				ESRD			
**Characteristic**	**Category**	**Adjusted HR (95% CI)**	***P* value**	**Characteristic**	**Category**	**Adjusted HR (95% CI)**	***P* value**
Stage	Stage 1	0.38 (0.08–1.89)	0.2351	Treatment	Dialysis	2.36 (2.23–2.50)	< 0.0001
	Stage 2	0.79 (0.40–1.54)	0.4898		Transplant	Reference	N/A
	Stage 3	Reference	N/A				
	Stage 4	1.59 (1.11–2.26)	0.0105				
	Stage 5	2.71 (1.32–5.53)	0.0063				
Age group	< 35 years	0.53 (0.03–8.44)	0.6567	Age group	< 18 years	0.15 (0.02–1.05)	0.0562
					18–24 years	0.19 (0.08–0.45)	0.0002
					25–34 years	0.18 (0.12–0.27)	< 0.0001
	35–44 years	0.79 (0.15–4.12)	0.7842		35–44 years	0.20 (0.16–0.24)	< 0.0001
	45–54 years	0.24 (0.05–1.28)	0.0952		45–54 years	0.28 (0.25–0.30)	< 0.0001
	55–64 years	0.89 (0.40–1.95)	0.7654		55–64 years	0.48 (0.45–0.52)	< 0.0001
	65–74 years	Reference	N/A		65–74 years	Reference	N/A
	75–84 years	1.70 (1.13–2.56)	0.0112		75–84 years	1.75 (1.62–1.90)	< 0.0001
	85 years and older	3.45 (2.28–5.23)	< 0.0001		85 years and older	2.77 (2.44–3.14)	< 0.0001
Sex	Female	0.76 (0.55–1.05)	0.0976	Sex	Female	0.91 (0.86–0.96)	0.0006
	Male	Reference	N/A		Male	Reference	N/A
Race	Black	1.20 (0.77–1.88)	0.4185	Race	Black	0.92 (0.85–1.00)	0.0492
	Hispanic	1.29 (0.37–4.49)	0.6910		Hispanic	0.71 (0.64–0.79)	< 0.0001
	Asian	1.55 (0.65–3.71)	0.3261		Asian	0.63 (0.52–0.76)	< 0.0001
	Other or unknown^ a^	0.57 (0.16–2.01)	0.3787		Other or unknown^ a^	0.88 (0.66–1.18)	0.4013
	White	Reference	N/A		White	Reference	N/A

*ADPKD* Autosomal dominant polycystic kidney disease, *CI* Confidence interval, *CKD* Chronic kidney disease, *ESRD* End-stage renal disease, *HR* hazard ratio, *N/A* Not applicable, *USRDS* United States Renal Data System

*Notes*: Adjusted HRs were estimated from the Cox model, including treatment, age group, sex, and race. Age and treatment were determined at study entry (start of patient-time at risk)

^a^In addition to “White,” “Black,” “Hispanic,” and “Asian” in the non-ESRD CKD USRDS dataset, “race” also included “Native American,” “Other,” and “Unknown,” which have been grouped in this table as “Other or unknown.” In addition to “White,” “Black/African American” (referred to as “Black” in this table), “Hispanic,” and “Asian” in the ESRD USRDS dataset, “race” also included “American Indian or Alaska Native,” “Native Hawaiian or Pacific Islander,” “Other or Multiracial,” and Unknown, which have been grouped in this table as “Other or unknown.”

Among the subset of patients aged ≥ 65 years and within the non-ESRD CKD cohort, patients in stage 4 CKD (HR = 1.60, *P* = 0.0096) had a statistically significantly higher risk of death than patients in stage 3 CKD (reference group) (Table 5). There was not a statistically significant difference in risk of death between patients in stage 5 CKD (HR = 1.85, *P* = 0.1837) and stage 3 CKD. Patients aged 75 to 84 years (HR = 1.70, *P* = 0.0108) and ≥ 85 years (HR = 3.43, *P* < 0.0001) had a statistically significantly higher risk of death than patients aged 65 to 74 years (reference group). There were no statistically significant differences in mortality risk between male and female patients or between racial groups in patients aged ≥ 65 years within the non-ESRD cohort.

**Table 5:** Adjusted hazard ratios among ADPKD patients aged 65 years and older

Non-ESRD CKD				ESRD			
**Characteristic**	**Category**	**Adjusted HR (95% CI)**	***P* value**	**Characteristic**	**Category**	**Adjusted HR (95% CI)**	***P* value**
Stage	Stage 1	0.28 (0.04–2.01)	0.2058	Treatment	Dialysis	1.92 (1.78–2.07)	< 0.0001
	Stage 2	0.71 (0.35–1.47)	0.3588		Transplant	Reference	N/A
	Stage 3	Reference	N/A				
	Stage 4	1.60 (1.12–2.29)	0.0096				
	Stage 5	1.85 (0.75–4.60)	0.1837				
Age group	65–74 years	Reference	N/A	Age group	65–74 years	Reference	N/A
	75–84 years	1.70 (1.13–2.55)	0.0108		75–84 years	1.83 (1.69–1.98)	< 0.0001
	85 years and older	3.43 (2.27–5.19)	< 0.0001		85 years and older	3.04 (2.67–3.46)	< 0.0001
Sex	Female	0.77 (0.55–1.07)	0.1178	Sex	Female	0.93 (0.87–1.00)	0.0419
	Male	Reference	N/A		Male	Reference	N/A
Race	Black	1.41 (0.90–2.21)	0.1377	Race	Black	0.87 (0.77–0.97)	0.0150
	Hispanic	1.22 (0.30–4.93)	0.7847		Hispanic	0.69 (0.60–0.80)	< 0.0001
	Asian	1.48 (0.60–3.65)	0.3925		Asian	0.71 (0.57–0.89)	0.0025
	Other or unknown^ a^	0.53 (0.13–2.14)	0.3709		Other or unknown^ a^	0.77 (0.51–1.17)	0.2228
	White	Reference	N/A		White	Reference	N/A

*ADPKD* Autosomal dominant polycystic kidney disease, *CI* Confidence interval, *CKD* Chronic kidney disease, *ESRD* End-stage renal disease, *HR* Hazard ratio, *N/A* Not applicable, *USRDS* United States Renal Data System

*Notes*: Adjusted HRs were estimated from the Cox model, including treatment, age group, sex, and race. Age and treatment were determined at study entry (start of patient-time at risk)

^a^ In addition to “White,” “Black,” “Hispanic,” and “Asian” in the non-ESRD CKD USRDS dataset, “race” also included “Native American,” “Other,” and “Unknown,” which have been grouped in this table as “Other or unknown.” In addition to “White,” “Black/African American” (referred to as “Black” in this table), “Hispanic,” and “Asian” in the ESRD USRDS dataset, “race” also included “American Indian or Alaska Native,” “Native Hawaiian or Pacific Islander,” “Other or Multiracial,” and Unknown, which have been grouped in this table as “Other or unknown.”

Among the subset of patients aged ≥ 65 years and within the ESRD cohort, patients receiving dialysis were statistically significantly more likely to experience death than patients who received a transplant (HR = 1.92, *P* < 0.0001) (Table 5). Patients aged 75 to 84 years (HR = 1.83, *P* < 0.0001) and ≥ 85 years (HR = 3.04, *P* < 0.0001) had a statistically significantly higher risk of death than patients aged 65 to 74 years (reference group). Female patients were statistically significantly less likely to experience death than male patients (HR = 0.93, *P* = 0.0419). Black (HR = 0.87, *P* = 0.0150), Hispanic (HR = 0.69, *P* < 0.0001), and Asian (HR = 0.71, *P* = 0.0025) patients had a statistically significantly lower risk of death than White patients (Table 5).

## Discussion

This retrospective study using US data provides further estimates of mortality risk and racial differences associated with mortality for patients with ADPKD who have progressed to ESRD and those who have not developed ESRD. For patients aged ≥ 65 years with ADPKD and ESRD, unadjusted mortality observed in this study was numerically lower than previously reported (approximately 10 per 100 vs. 16 per 100 patient-years [16]). In our study, after standardizing to the US population age distribution, ADPKD ESRD mortality for patients aged ≥ 65 years was approximately 13 per 100 patient-years. The numerically lower mortality rate in this study compared with Reule et al. [16] might be partially due to our use of more recent data and may reflect improvements in the treatment and management of ADPKD and/or ESRD, as well as related end-organ complications, including cardiovascular disease. The difference between ADPKD ESRD mortality in patients aged ≥ 65 years in this study (99.8 per 1,000 patient-years) and ESRD mortality in general in the US (216 per 1,000 patient-years) [27], both unadjusted for age, is perhaps due to the number of non-ADPKD patients with ESRD who have diabetes [27], a major cause of premature mortality [28].

We also found racial differences in mortality in both non-ESRD CKD and ESRD cohorts, and in mortality risk in the ESRD cohort, among patients aged ≥ 65 years with ADPKD. Our results from the Cox modeling suggest that Black patients have a lower risk of death compared with White patients aged ≥ 65 years with ADPKD and ESRD. Age-adjusted results among patients with ADPKD and aged ≥ 65 years suggest that mortality may be greatest among Black patients with non-ESRD CKD and greatest among White patients with ESRD. This disparity may be explained by a survivorship bias. Black patients may be less likely than other racial groups to survive long enough to reach ESRD, perhaps because of inequities in care (e.g., access to healthcare, health literacy, physician biases) [4]. ADPKD also may be underdiagnosed in Black patients with a hypertension comorbidity [4], potentially leading to lack of treatment for ESRD in these patients. Black patients who do survive may be healthier, thereby explaining the reversal in the patterns of age-adjusted survivorship between Black and White patients. The suggestion of a potential survivorship bias in the findings by race warrants further research.

The analyses by CKD stage depend on the accuracy of staging. There is some normal biologic variability in the glomerular filtration rate and natural variability in the manifestation of disease (e.g., the estimated glomerular filtration rate at any given timepoint), as well as variability in assessment of stage of CKD due to test accuracy. When ADPKD mortality is studied by stage, the USRDS dataset may be most informative for patients aged ≥ 65 years, given that the non-ESRD CKD dataset available from the USRDS is derived from Medicare claims data for patients aged ≥ 65 years. Accordingly, the findings would be representative of patients aged ≥ 65 years with non-ESRD CKD, although a study limitation is that the findings for non-ESRD CKD may not be generalizable to the overall population of patients with ADPKD and non-ESRD CKD. Additionally, several subgroups in the non-CKD ESRD dataset had relatively few patients, leading to imprecise estimates of mortality risk. Future research is warranted to explore mortality among patients younger than 65 years with non-ESRD ADPKD using another database representative of this population. However, the USRDS dataset provided robust data for the ESRD cohort, regardless of patient age, because this dataset includes nearly the entire US ESRD population [29, 30].

## Conclusions

Using more recently available data from USRDS that included the entire population of patients with ESRD in the US and patients diagnosed with CKD within the Medicare 5% database, we estimated mortality rates specific to patients with ADPKD overall and by age, sex, and race. Mortality rates specific to patients aged ≥ 65 years with ADPKD and ESRD are lower than previously reported estimates and suggest improved survival, perhaps due to more effective treatment and disease management. The results of this study suggest a lower risk of death for communities of color with ADPKD and ESRD than for White patients, based on comparison of mortality rates in ESRD and non-ESRD cohorts. These data also suggest potential racial differences in mortality among patients aged ≥ 65 years with ADPKD in both non-ESRD CKD and ESRD cohorts, and a possible survivorship effect among Black patients aged ≥ 65 years with ADPKD. These findings fill an important gap in the literature on ADPKD mortality in the US.

### Supplementary Information

**Additional file 1: Table S1.** Mortality among patients with ADPKD overall, by sex, and by race. **Table S2.** Mortality among patients with ADPKD aged ≥ 65 years overall, by sex, and by race.

## Data Availability

The data reported here have been supplied by the USRDS. The interpretation and reporting of these data are the responsibility of the author(s) and in no way should be seen as an official policy or interpretation of the US government. To request data, please refer to the USRDS website at https://www.usrds.org/for-researchers/.

## Abbreviations

ADPKD: Autosomal dominant polycystic kidney disease
CI: Confidence interval
CKD: Chronic kidney disease
CMS: Centers for Medicare & Medicaid Services
ESRD: End-stage renal disease
HR: Hazard ratio
ICD-9-CM: International Classification of Diseases, Ninth Edition, Clinical Modification
ICD-10-CM: International Classification of Diseases, Tenth Edition, Clinical Modification
SAF: Standard Analytic File (CMS)
SD: Standard deviation
UNOS: United Network for Organ Sharing
US: United States
USRDS: United States Renal Data System

## References

[1] Tan YC, Blumenfeld J, Rennert H (2011). Autosomal dominant polycystic kidney disease: genetics, mutations and microRNAs. Biochim Biophys Acta.

[2] Chapman AB (2008). Approaches to testing new treatments in autosomal dominant polycystic kidney disease: insights from the CRISP and HALT-PKD studies. Clin J Am Soc Nephrol.

[3] Blanchette CM, Liang C, Lubeck DP (2015). Progression of autosomal dominant kidney disease: measurement of the stage transitions of chronic kidney disease. Drugs Context.

[4] Murphy EL, Dai F, Blount KL (2019). Revisiting racial differences in ESRD due to ADPKD in the United States. BMC Nephrol.

[5] Benjamin O, Lappin SL. End-stage renal disease. 2022. Available at: https://www.ncbi.nlm.nih.gov/books/NBK499861/. Accessed 28 Feb 2022

[6] Roumelioti ME, Argyropoulos C, Buysse DJ, Nayar H, Weisbord SD, Unruh ML (2010). Sleep quality, mood, alertness and their variability in CKD and ESRD. Nephron Clin Pract.

[7] Weisbord SD, Fried LF, Arnold RM (2005). Prevalence, severity, and importance of physical and emotional symptoms in chronic hemodialysis patients. J Am Soc Nephrol.

[8] Pisoni RL, Wikstrom B, Elder SJ (2006). Pruritus in haemodialysis patients: international results from the Dialysis Outcomes and Practice Patterns Study (DOPPS). Nephrol Dial Transplant.

[9] Chen SS, Al Mawed S, Unruh M (2016). Health-related quality of life in end-stage renal disease patients: how often should we ask and what do we do with the answer?. Blood Purif.

[10] Abdel-Kader K, Jhamb M, Mandich LA (2014). Ecological momentary assessment of fatigue, sleepiness, and exhaustion in ESKD. BMC Nephrol.

[11] Zelmer JL (2007). The economic burden of end-stage renal disease in Canada. Kidney Int.

[12] Wang V, Vilme H, Maciejewski ML, Boulware LE (2016). The economic burden of chronic kidney disease and end-stage renal disease. Semin Nephrol.

[13] Moreno Velasquez I, Tribaldos Causadias M, Valdes R (2019). End-stage renal disease-financial costs and years of life lost in Panama: a cost-analysis study. BMJ Open.

[14] United States Renal Data System. 2021 USRDS annual data report: epidemiology of kidney disease in the United States. 2021. Available at: https://adr.usrds.org/2021. Accessed 4 Mar 2022 10.1053/j.ajkd.2022.02.001 PMC8935019 35331382

[15] United States Renal Data System. 2020 USRDS annual data report, volume 2: end stage renal disease. 2020. https://adr.usrds.org/2020/end-stage-renal-disease/5-mortality. Accessed 7 June 2021.

[16] Reule S, Sexton DJ, Solid CA, Chen SC, Collins AJ, Foley RN (2014). ESRD from autosomal dominant polycystic kidney disease in the United States, 2001–2010. Am J Kidney Dis.

[17] Prakash S, Rodriguez RA, Austin PC (2010). Racial composition of residential areas associates with access to pre-ESRD nephrology care. J Am Soc Nephrol.

[18] Smith SR, Svetkey LP, Dennis VW (1991). Racial differences in the incidence and progression of renal diseases. Kidney Int.

[19] Ku E, Yang W, McCulloch CE (2020). Race and mortality in CKD and dialysis: findings from the Chronic Renal Insufficiency Cohort (CRIC) study. Am J Kidney Dis.

[20] Centers for Medicare & Medicaid Services. CMS 2728. ESRD medical evidence report medicare entitlement and/or patient registration. 2006. Available at: https://www.cms.gov/Medicare/CMS-Forms/CMS-Forms/CMS-Forms-Items/CMS008867. Accessed 7 Mar 2022

[21] Centers for Medicare & Medicaid Services. CMS 2746. ESRD death notification. 2006. Available at: https://www.cms.gov/Medicare/CMS-Forms/CMS-Forms/CMS-Forms-Items/CMS008869. Accessed 7 Mar 2022.

[22] United States Renal Data System. 2020 researcher’s guide to the USRDS database. 2020. Available at: https://www.usrds.org/media/2482/2020_usrds_researcher_guide.pdf. Accessed 28 Feb 2022.

[23] Dobson AJ, Kuulasmaa K, Eberle E, Scherer J (1991). Confidence intervals for weighted sums of Poisson parameters. Stat Med.

[24] United States Census Bureau. Current population survey, annual social and economic supplement. 2019. Age and sex composition in the United States: 2019. 2021. Available at: https://www.census.gov/data/tables/2019/demo/age-and-sex/2019-age-sex-composition.html. Accessed 30 Nov 2021.

[25] Firth D (1993). Bias reduction of maximum likelihood estimates. Biometrika.

[26] Roberts AW, Ogunwole SU, Blakeslee L, Rabe MA. The population 65 years and older in the United States: 2016. 2018. Available at: https://www.census.gov/content/dam/Census/library/publications/2018/acs/ACS-38.pdf. Accessed 7 Mar 2022.

[27] United States Renal Data System. 2018 USRDS annual data report, Volume 2: ESRD in the United States. 2018. Available at: https://www.usrds.org/media/2283/2018_volume_2_esrd_in_the_us.pdf. Accessed 4 Mar 2022

[28] Heron M (2021). Deaths: leading causes for 2019. Natl Vital Stat Rep.

[29] Foley RN, Collins AJ (2013). The USRDS: what you need to know about what it can and can't tell us about ESRD. Clin J Am Soc Nephrol.

[30] Foster BJ, Mitsnefes MM, Dahhou M, Zhang X, Laskin BL (2018). Changes in excess mortality from end stage renal disease in the United States from 1995 to 2013. Clin J Am Soc Nephrol.

