# Supplementary material for: Mortality risk in patients with autosomal dominant polycystic kidney disease
Source: BMC Nephrol. 2024 Feb 16;25:56. doi: 10.1186/s12882-024-03484-3 (PMC10870477; doi:10.1186/s12882-024-03484-3)
Supplement: Supplementary file 1 — Additional file 1: Table S1. Mortality among patients with ADPKD overall, by sex, and by race. Table S2. Mortality among patients with ADPKD aged ≥ 65 years overall, by sex, and by race. [file 12882_2024_3484_MOESM1_ESM.docx]

# Supplementary Information

Additional File 1: Table S1. Mortality among patients with ADPKD overall, by sex, and by race. Table S2. Mortality among patients with ADPKD aged ≥ 65 years overall, by sex, and by race.

Table S1. Mortality Among Patients With ADPKD Overall, by Sex, and by Race

| Characteristics | Non-ESRD CKD | | ESRD | |
| --- | --- | --- | --- | --- |
|  | Mortality (95% CI) | Age-adjusted mortality (95% CI) | Mortality (95% CI) | Age-adjusted mortality (95% CI) |
| Overall | 65.6  (57.6-74.4) | 18.4  (13.3-23.5) | 53.9 (52.4-55.4) | 37.4  (32.5-42.2) |
| By age group | | | | |
| < 18 years | —^a^ | N/A | 10.1 (0.3-56.5) | N/A |
| 18-24 years | —^a^ | N/A | 14.2 (4.6-33.1) | N/A |
| 25-34 years | 0.0 (0.0-80.4)^a^ | N/A | 15.5 (10.1-22.7) | N/A |
| 35-44 years | 14.9 (0.4-82.8) | N/A | 18.5 (15.5-21.9) | N/A |
| 45-54 years | 9.1 (1.1-32.8) | N/A | 24.1 (22.1-26.2) | N/A |
| 55-64 years | 39.4  (20.3-68.8) | N/A | 39.1 (37.0-41.3) | N/A |
| 65-74 years | 37.9  (28.3-49.6) | N/A | 78.4 (74.7-82.3) | N/A |
| 75-84 years | 78.9  (63.4-97.1) | N/A | 161.9 (152.4-171.8) | N/A |
| ≥ 85 years | 159.0  (127.0-196.6) | N/A | 327.5 (291.0-367.3) | N/A |
| By sex | | | | |
| Male | 76.9  (65.0-90.3) | 22.2  (12.1-32.2) | 54.7 (52.6-56.8) | 36.6  (34.1-39.1) |
| Female | 53.1  (42.9-65.1) | 15.0  (10.0-20.0) | 53.0 (50.8-55.2) | 38.1  (28.7-47.4) |
| By race | | | | |
| White | 64.2  (55.4-73.9) | 20.9  (13.3-28.5) | 55.7 (54.0-57.5) | 41.1  (30.9-51.4) |
| Black | 79.7  (56.4-109.4) | 17.1  (10.8-23.5) | 58.4 (54.0-63.1) | 39.1  (33.3-44.9) |
| Hispanic | 37.7  (4.6-136.2) | 6.4 (0-15.3) | 39.2 (35.4-43.2) | 27.1  (23.4-30.9) |
| Asian | 90.0  (29.2-210.0) | 10.1 (1.0-19.2) | 43.6 (35.8-52.6) | 21.8  (17.5-26.1) |
| Other or unknown^b^ | 40.9  (11.2-104.8) | 9.7 (0-19.6) | 50.4 (37.1-67.1) | 38.2  (17.4-59.0) |

ADPKD = autosomal dominant polycystic kidney disease; CI = confidence interval; CKD = chronic kidney disease; ESRD = end-stage renal disease; N/A = not applicable; USRDS = United States Renal Data System.

^a^ Results for the non-ESRD CKD cohort are provided for patients aged < 35 years at study entry as a group due to small sample sizes.

^b^ In addition to “White,” “Black,” “Hispanic,” and “Asian” in the non-ESRD CKD USRDS dataset, “race” also included “Native American,” “Other,” and “Unknown,” which have been grouped in this table as “Other or unknown.” In addition to “White,” “Black/African American” (referred to as “Black” in this table), “Hispanic,” and “Asian” in the ESRD USRDS dataset, “race” also included “American Indian or Alaska Native,” “Native Hawaiian or Pacific Islander,” “Other or Multiracial,” and “Unknown,” which have been grouped in this table as “Other or unknown.”

## Table S2. Mortality Among Patients With ADPKD Aged ≥ 65 Years Overall, by Sex, and by Race

| Characteristic | Non-ESRD CKD | | ESRD | |
| --- | --- | --- | --- | --- |
|  | Mortality (95% CI) | Age-adjusted mortality (95% CI) | Mortality (95% CI) | Age-adjusted mortality (95% CI) |
| Overall | 74.2 (64.9-84.5) | 61.9 (53.4-70.4) | 99.8 (96.4-103.3) | 129.6 (124.2-135.0) |
| By age group | | | | |
| 65-74 years | 35.2 (25.7-47.2) | N/A | 68.8  (65.5-72.2) | N/A |
| 75-84 years | 72.3 (57.8-89.5) | N/A | 158.9  (150.1-168.2) | N/A |
| ≥ 85 years | 159.7 (129.5-194.8) | N/A | 340.6  (307.6-376.2) | N/A |
| By sex | | | | |
| Male | 85.7 (72.2-101.1) | 72.2 (59.5-85.0) | 104.4  (99.5-109.6) | 134.4  (126.7-142.1) |
| Female | 60.8 (48.6-75.1) | 50.4 (39.3-61.5) | 95.3 (90.6-100.2) | 124.8  (117.2-132.5) |
| By race | | | | |
| White | 70.3 (61.4-81.4) | 59.5 (50.3-68.8) | 101.7 (97.8-105.8) | 136.1  (129.6-142.7) |
| Black | 107.5 (75.7-148.1) | 82.7 (55.1-110.2) | 104.7 (94.2-116.0) | 123.3  (109.1-137.4) |
| Hispanic | 57.2 (6.9-206.5) | 41.4 (0-98.9) | 80.0  (69.6-91.5) | 100.3  (83.4-117.1) |
| Asian | 95.5 (31.0-222.7) | 51.2 (4.6-97.8) | 91.4 (73.1-112.9) | 102.9  (79.6-126.3) |
| Other or unknown^a^ | 49.5 (13.5-126.8) | 54.2 (0-108.4) | 86.1 (55.1-128.1) | 94.2  (53.4-135.1) |

ADPKD = autosomal dominant polycystic kidney disease; CI = confidence interval; CKD = chronic kidney disease; ESRD = end-stage renal disease; N/A = not applicable; USRDS = United States Renal Data System.

^a^ In addition to “White,” “Black,” “Hispanic,” and “Asian” in the non-ESRD CKD USRDS dataset, “race” also included “Native American,” “Other,” and “Unknown,” which have been grouped in this table as “Other or unknown.” In addition to “White,” “Black/African American” (referred to as “Black” in this table), “Hispanic,” and “Asian” in the ESRD USRDS dataset, “race” also included “American Indian or Alaska Native,” “Native Hawaiian or Pacific Islander,” “Other or Multiracial,” and “Unknown,” which have been grouped in this table as “Other or unknown.”
